# Preliminary experience in using the lateral single-incision laparoscopic totally extraperitoneal approach for inguinal hernia repair

**DOI:** 10.1007/s13304-024-02058-0

**Published:** 2024-12-18

**Authors:** Yizhong Zhang, Weidong Wu, Junjie Chen, Xianke Si, Jian Li, Tingfeng Wang

**Affiliations:** 1 Department of General Surgery, Shanghai Pudong Hospital, Fudan University Pudong Medical Center, 2800 Gongwei Road, Pudong, Shanghai, 201399 China; 2 Department of Hernia and Hepatobiliary Surgery, the First Affiliated Hospital of Ningbo University, 247 Renmin Road, Ningbo, 31500 China; 3 Gastrointestinal Surgery Department of General Surgery Center, Shanghai General Hospital, Shanghai Jiao Tong University School of Medicine, 85 Wujin Road, Shanghai, 200080 China; 4 Department of General Surgery, Ningbo Beilun District Traditional Chinese Medicine Hospital, 221 Yanan Xi Road, Shanghai, 200041 China; 5 Department of Hernia and Abdominal Wall Surgery, Putuo Hospital Affiliated to Shanghai University of TCM, Shanghai, 200062 China; 6 Department of General Surgery, Yangpu Hospital, School of Medicine, Tongji University, 450 Tengyue Road, Yangpu, Shanghai, 200090 China

**Keywords:** L-SILTEP, Inguinal hernia, Feasibility, Safety, Effectiveness

## Abstract

To evaluate the feasibility, safety, and efficacy of the lateral single-incision laparoscopic totally extraperitoneal (L-SILTEP) approach in patients with inguinal hernia who had contraindications to the midline approach. This study included 58 patients who underwent L-SILTEP. Data on their baseline characteristics and perioperative details were collected. Quality of life and cosmetic satisfaction assessments were performed. Of the evaluated patients, 25.9% had a history of middle and lower abdominal surgery and 10.3% had skin diseases around the umbilicus. The mean surgical duration, blood loss volume, and incision length were 53.5 (± 22.3) min, 7.2 (± 9.7) mL, and 2.0 (± 0.13) cm, respectively. Additionally, 29.3% of patients experienced intraoperative peritoneal rupture, and one patient had epigastric vessel bleeding. The 6-, 24-, and 48-h postoperative pain scores were 3.0 (± 0.6), 1.6 (± 0.6), and 1.1 (± 0.4), respectively. Postoperative complications included seroma (*n* = 3), hematoma (*n* = 1), and scrotal edema (*n* = 1). The surgical incision in the L-SILTEP approach was more aesthetically pleasing than that in previous surgeries. Approximately 17.2%, 8.6%, and 10.3% of patients reported pain, mesh sensation, and movement limitation, respectively. Severe or disabling symptoms were not reported, and there were no cases of 30-day readmissions. Hernia recurrence or incisional hernia was not observed over a mean follow-up duration of 14.6 (± 6.1) months. L-SILTEP can be used for patients with contraindications to the midline approach. Furthermore, it is a safe and effective procedure.

## Introduction

To further improve minimally invasive methods, single-incision laparoscopic surgery (SILS) was developed by surgeons. Compared with conventional multiport (MP) approaches, SILS offers advantages in terms of being less invasive and more aesthetically pleasing. With the use of commercial ports, SILS technology is widely employed across various specialties, such as general surgery, bariatric surgery, urology, and gynecology [1–5]. In recent years, the number of surgeries performed using SILS has been increasing [6–10].

In 2009, Cugura et al. first reported the single-incision laparoscopic totally extraperitoneal (SIL-TEP) approach [11]. According to a newly published systematic review and meta-analysis [12], the SIL-TEP approach is more popular than the single-incision laparoscopic transabdominal preperitoneal approach (SIL-TAPP). Moreover, its safety and efficacy are similar to those of MP-TEP. In SIL-TEP, a subumbilical incision and central approach are commonly used, which can be challenging if this region has been utilized in a previous surgery. Meanwhile, medical issues such as periumbilical infection and eczema should also be considered when selecting the surgical channel site.

To address the abovementioned issues, the lateral single-incision laparoscopic totally extraperitoneal (L-SILTEP) approach, a laparoscopic technique that utilizes lateral abdominal access, was innovatively introduced [13]. This study aimed to conduct a preliminary review to assess the safety and efficacy of the L-SILTEP approach in patients with a history of middle and lower abdominal surgery.

## Methods

### Study design

This retrospective study enrolled 58 patients who underwent surgery using the L-SILTEP approach for inguinal hernia repair between June 2021 and April 2023. All procedures were performed by skilled surgeons using a 10 × 15 cm lightweight polypropylene mesh without fixation. The study included patients from three hernia centers: Shanghai General Hospital, Shanghai (*n* = 16); Affiliated Hospital of the Medical School of Ningbo University, Ningbo (*n* = 34); and Fudan University Pudong Medical Center, Shanghai (*n* = 8). Informed consent was obtained from all participants. The study protocol was reviewed and approved by the ethics committees of the three hospitals, with approval numbers 2020-053, XJS20191206, and 2020PWR-03 respectively.

Data on patient characteristics, including age, sex, body mass index, American Society of Anesthesiologists (ASA) classification score, and hernia features, were collected. Perioperative data included incision length, surgical duration, blood loss volume, and incidence rates of peritoneal rupture and complications (e.g., vascular, vas deferens, bowel, and bladder injury). Postoperative data included pain scores assessed using the visual analog scale, incidence rates of complications (e.g., surgical site infection, mesh infection, seroma, hematoma, and scrotal edema), length of hospital stay, incisional hernia rate, and recurrence rate.

### Patient selection

The standardized preoperative workup of patients began with a detailed history and physical examination. The inclusion criteria were as follows: (1) patients with unilateral inguinal hernia who consented to L-SILTEP, (2) those aged 18–80 years, (3) and those with preoperative ASA grade 1 or 2, with a preference for patients with a history of middle and lower abdominal surgery. The exclusion criteria were as follows: (1) patients with acute incarcerated hernia, (2) those with a body mass index of > 30 kg/m^2^, (3) those with cardiopulmonary complications preventing tolerance of general anesthesia and surgery, and (4) those with surgical scar or skin infection near the McBurney point or the anti-McBurney point.

### Operative technique

After the induction of general anesthesia, the patient was placed in the Trendelenburg position at a 15° head-down tilt, and the operating table was tilted 15° to the healthy side. The chief surgeon stood on the same side as the hernia, with the first assistant positioned behind the chief surgeon next to the patient’s shoulder. Urinary catheterization was routinely performed. The pneumoperitoneum pressure was set to 12 mmHg, and the pneumoperitoneum flow rate was set at 20 L/min.

The six-step L-SILTEP approach is as follows:

Step 1: Incision selection and device placement

**Fig. 1:**
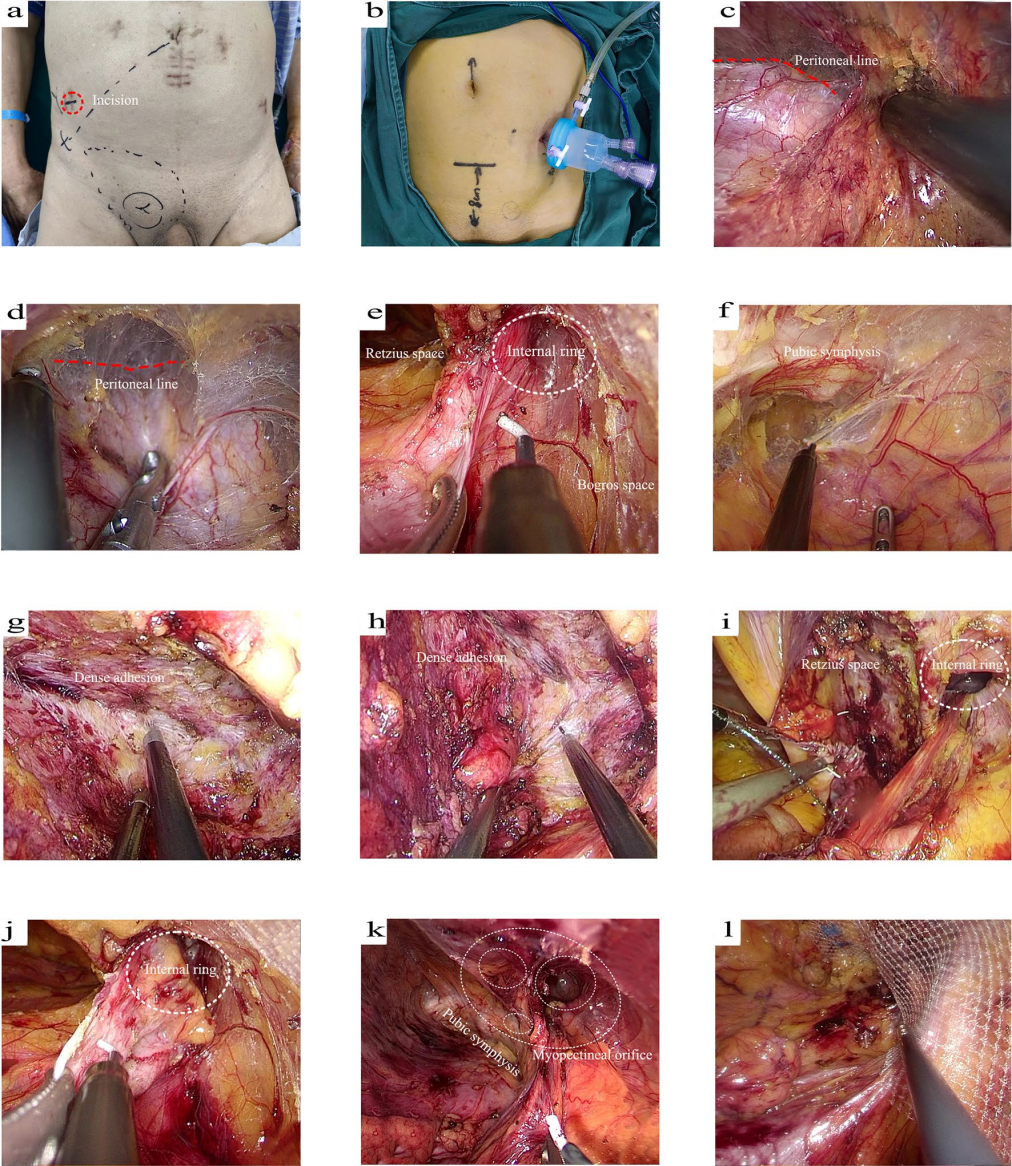
L-SILTEP procedure. **a** Selection of incision location and **b** placement of port. **c** Freeing the extraperitoneal space above the internal ring. **d** and **e**: Exposure of the Bogros space and internal ring. **f** Exposure of the Retzius space. **g** and **h** Separation of dense adhesions. **i** Closure of the peritoneum by continuous suturing. **j** Dissection of the hernia sac. **k** Exposure of the entire myopetinal orifice. **l** Placement of the mesh

Access was gained via a 2–2.2-cm transverse incision made 1–2 cm above the McBurney point or the anti-McBurney point (Fig. 1a). Subsequently, the aponeurosis of the external oblique abdominal muscle was cut anterogradely, and the internal oblique and transverse abdominal muscles were separated by pulling perpendicular to the muscle fibers to expose the extraperitoneal fat. After enlarging the preperitoneal space with the index finger, the port was inserted to establish the operative space (Fig. 1b).

Step 2: Establishment of the preperitoneal space

Establishment of the preperitoneal space involved freeing the extraperitoneal space above the internal ring (Fig. 1c), followed by exposing the inner ring and expanding into the Bogros space, guided by the peritoneum (Fig. 1d, e). Subsequently, medial turning and dissection into the Retzius space were performed until reaching the pubic symphysis and crossing the midline (Fig. 1f). In cases of dense adhesions from previous surgeries, careful separation close to the abdominal wall was necessary (Fig. 1g, h). If required, the peritoneum was actively incised and extended inward across the adhesive area, followed by closure with a running suture (Fig. 1i). The space was then expanded from bottom to top, up to 8 cm above the pubic symphysis and 2–3 cm below the Cooper’s ligament.

Step 3: Hernia sac dissection

Using an indirect hernia as an example, the outer and posterior parts of the hernia sac were initially dissociated from the spermatic cord, guided by the peritoneum. Subsequently, the hernia sac was laterally pulled to dissociate the anteromedial part. The technical approach to hernia sac dissection emphasized starting laterally and maintaining a peritoneal orientation (Fig. 1j). In cases where the sac was excessive or densely adherent, it was ligated and transected below the inner ring to avoid damaging the spermatic cord structures. In female patients, the ligamentum teres uteri was preserved by longitudinally cutting the hernia sac below the inner ring, ensuring a distance of > 5 cm from it. Finally, the peritoneum was closed using a running suture with barbed sutures.

Step 4: Parietalization of the spermatic cord and space expansion

The hernia sac and peritoneum were completely detached from the cord, a process known as parietalization. The Bogros space was further extended along the medial surface of the iliopubic tract to the position of the anterior superior iliac spine. Following dissection, the entire myopetinal orifice was cleared of peritoneal and fatty tissues (Fig. 1k).

Step 5: Mesh placement and preperitoneal deflation under vision

For hernia repair, either the 3DMax lightweight mesh in a large size (Bard) or the lightweight mesh (Johnson & Johnson UMF) was used. The mesh was inserted through the port and expanded around the hernia ring to completely cover the myopetinal orifice. Subsequently, the lower edge of the mesh was secured with a clamp to prevent displacement during preperitoneal deflation (Fig. 1l).

Step 6: Removal of the port and closure of the incision

The port was removed, and the fascia of the internal oblique muscle was sutured. Subsequently, the incised external oblique aponeurosis was sutured continuously. Finally, the skin incision was closed with an absorbable suture.

### Quality of life and cosmetic satisfaction assessment

Quality of life and cosmetic satisfaction assessments were conducted either in person, via telephone, or through electronic communication between the clinical team and patients who consented to participate in the study and data collection. Quality of life was evaluated using the Carolina Comfort Scale (CCS), a validated hernia-specific questionnaire with a rating scale of 0–5 (0 indicating “no symptoms” and 5 indicating “disabling symptoms”), assessing pain, mesh sensation, and movement limitation. Cosmetic satisfaction was assessed using the Score of Access-Site Satisfaction & Consideration questionnaire, where satisfaction levels were rated on a scale of 1–10: 1 for extreme dissatisfaction, 5 for neutral satisfaction, and 10 for extreme satisfaction [14].

### Statistical analysis

Data analysis was performed using Statistical Package for the Social Sciences software version 22.0 (IBM Corporation, Armonk, NY, USA). Qualitative data are expressed as percentages, whereas quantitative data are expressed as mean ± standard deviation or median (min–max) depending on their distribution. Statistical comparisons between groups were conducted using the Wilcoxon rank-sum test for quantitative variables and Pearson’s chi-square test for categorical variables. A *P* value of < 0.05 was considered statistically significant.

## Results

This study included 58 patients, including 21 (36.2%) with a history of middle and lower abdominal surgery or umbilical skin disease. Table 1 presents the baseline characteristics of the patients. The mean surgical duration, blood loss volume, and incision length were 52 (21–117) min, 2 (2–40) mL, and 2.0 (± 0.13) cm, respectively. Intraoperative peritoneal rupture occurred in 17 (29.3%) patients. The incidence of peritoneal rupture in patients with and without surgical history was 46.7% and 23.3%, respectively, with no significant difference observed (p = 0.1658). One patient experienced intraoperative epigastric vessel bleeding, which was successfully managed with a clip. Postoperative pain scores at 6, 24, and 48 h, assessed using the visual analog scale, were 3.0 (± 0.6), 1.6 (± 0.6), and 1.1 (± 0.4), respectively. Five (8.6%) patients experienced postoperative complications (seroma, *n* = 3; preperitoneal space hematoma, *n* = 1; and scrotal edema, *n* = 1) that were managed conservatively. No hernia recurrence or incisional hernia was observed over a mean follow-up duration of 16 (6–25) months.

**Table 1:** Patient demographics

Characteristic	Value
Total number	58
Gender	
Male	51(87.9%)
Female	7 (12.1%)
Age (year)	58.5 (26–89)
BMI (kg/m^2^)	23.0 (± 2.4)
ASA	
1	37 (63.8%)
2	21 (36.2%)
Hernia side	
Right	37(63.8%)
Left	21(36.2%)
Hernia type (modified gilbert classification)	
II	19 (32.8%)
III	28 (48.3%)
IV	3 (5.2%)
V	4 (6.9%)
VI	2 (3.4%)
VII	2 (3.4%)
Recurrent hernia	0(0%)
Comorbidity	
Hypertension	6 (10.3%)
Type 2 Diabetes	7 (12.1%)
Hyperlipemia	3 (5.2%)
COPD	1 (1.7%)
History of middle and lower abdominal surgery	15 (25.9%)
Skin disease around umbilicus	6 (10.3%)

Table 2 shows the cosmetic satisfaction between patients who underwent previous surgeries and those who underwent surgery using the L-SILTEP approach. Overall, 21 patients with a history of surgery consistently reported that the incision in the current surgery was more aesthetically pleasing than that in the previous one, and the results were statistically significant (*p* < 0.001).

**Table 2:** Comparison of cosmetic satisfaction between previous surgery and L-SILTEP

Patient no	Previous surgery (PS)	Rating of PS	Rating of L-SILTEP	*P* value
1	Open inguinal hernia repair	4	10	< 0.001
2	Open inguinal hernia repair	5	10	
3	Caesarean section	1	9	
4	Open salpingectomy	2	10	
5	Open inguinal hernia repair	4	10	
6	Open resection of ileocecal stromal tumor	4	9	
7	Open radical gastrectomy	1	10	
8	Open radical prostatectomy	2	10	
9	Laparoscopic pancreaticoduodenectomy	3	10	
10	SIL umbilical hernia repair	6	8	
11	Open inguinal hernia repair	4	10	
12	Open umbilical hernia repair	4	7	
13	Open radical prostatectomy	5	8	
14	Open radical proctectomy	3	7	
15	Laparoscopic radical prostatectomy	7	8	
16	Laparoscopic radical prostatectomy	7	8	
17	Open small intestine resection	4	8	
18	Robotic radical prostatectomy	5	8	
19	Open cystolithotomy	3	8	
20	Laparoscopic radical prostatectomy	3	7	
21	Open right nephrectomy	6	8	

**Fig. 2:**
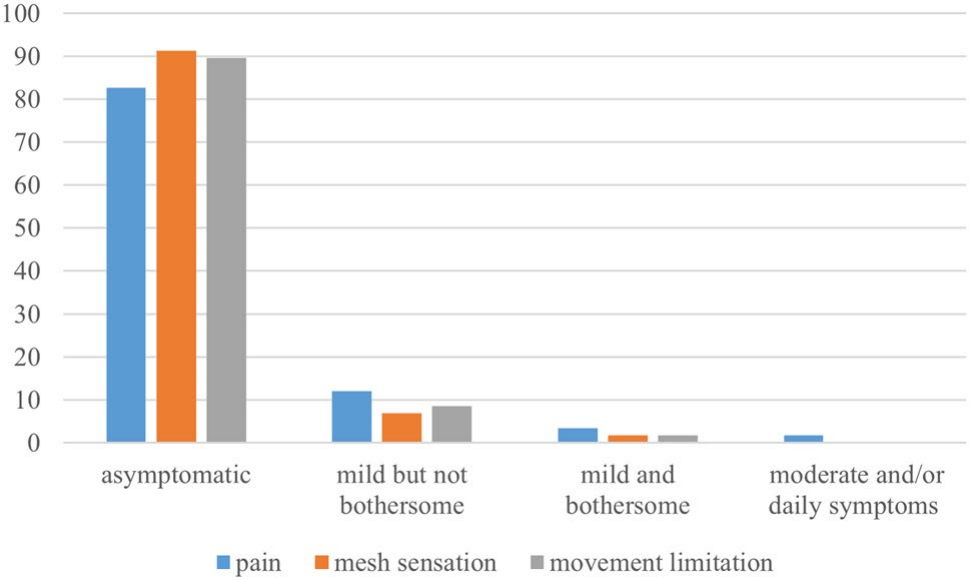
Percentage of patients categorized by severity grades for mesh-related symptoms

The majority of patients (77.6%) were asymptomatic after a median follow-up of 16 (6–25) months. Pain, mesh sensation, and movement limitation were reported by 17.2%, 8.6%, and 10.3% of patients, respectively. However, these symptoms were typically mild and not bothersome, and none of the patients experienced severe or disabling symptoms (Fig. 2).

## Discussion

The SILS approach for inguinal hernia repair has garnered attention in recent years. However, implementing laparoscopic surgery via the umbilical approach and managing postoperative recovery can be challenging due to surgical scars around the umbilicus or anterior lower abdominal wall, as well as skin conditions such as umbilical infection and eczema. To address these challenges, the L-SILTEP approach was innovatively adopted [13]. This method combines the advantages of SIL and serves as a beneficial complement to the central approach. In our study, all L-SILTEP cases were successfully performed. Preliminary data indicate that this technique is safe and effective, with high patient satisfaction.

The primary objective of implementing the lateral approach was to avoid encountering surgical scarring from previous procedures, such as middle and lower abdominal surgeries, at the beginning of the procedure, thereby allowing the procedure to begin in a normal anatomical setting. For example, there is an increased incidence of inguinal hernias following radical prostatectomy [15], and surgical scarring can significantly increase the difficulty and complication rates of herniorrhaphy using the midline approach [16, 17]. In this study, data from the lateral approach showed that the mean surgical duration, blood loss volume, incidence rate of perioperative complications, and postoperative pain scores were comparable to those reported in previous studies [18–21]. Additionally, the majority of patients who completed the CCS questionnaire were asymptomatic after a median follow-up of 16 (6–25) months. Patients commonly reported mild and non-bothersome symptoms, with none experiencing severe or disabling symptoms, similar to findings from MP-TAPP [22]. These findings suggest that the lateral approach reduces surgical complexity and enhances safety to a certain extent.

The main operational difficulties in the L-SILTEP approach are primarily peritoneal protection and preventing rupture. In this study, 17 patients experienced peritoneal rupture, resulting in an incidence rate of 29.3%, which is higher compared to the midline approach [14, 23]. In 12 cases, peritoneal rupture occurred at the intersection of the semilunar and semicircular lines. This was primarily due to an exceptionally thin peritoneum and tight adhesion between the preperitoneal and transverse abdominal fascia. Another contributing factor to the high incidence of peritoneal damage was dense adhesions caused by previous surgical scars. Damage to the peritoneum can lead to its flapping and lifting, which narrows the operating space and complicates surgery. Preventive measures included initial separation of the Bogros and Retzius spaces to enlarge the lower abdominal area. Subsequently, the upward separation of the semilunar and semicircular lines was performed to limit surgical impact, even in cases of peritoneal damage. Additionally, a left-hand controlled clamp was used to press the peritoneum downward, maintaining tension for optimal visibility. The right-hand controlled electrocoagulation hook was employed to gradually and precisely separate fused fascia or peritoneum, thus reducing the risk of further peritoneal damage.

With the advancement of this technology, we have discovered several unique advantages of the L-SILTEP approach. It effectively addresses challenges encountered in SILS procedures. First, it provides a clearer perspective for assessing the positional association between the hernia sac and the spermatic cord, offering a more ergonomic operating angle during sac dissection. This technical advantage is particularly beneficial for managing scrotal hernias. Second, after incising the extraperitoneal fat, direct access to the Bogros space becomes feasible. The creation and expansion of the operative space are progressively facilitated by pneumoperitoneal pressure under direct visualization. Subsequently, following the peritoneum to the inner ring simplifies the procedure by circumventing the intricate abdominal transverse fascia in the middle lower abdomen. Approximately 25.9% of patients had a history of lower abdominal surgery. However, surgical complexity could be effectively managed by surgeons skilled in utilizing the L-SILTEP approach.

Additionally, the L-SILTEP approach is associated with a reduced incidence of postoperative recurrence, incisional infection, and incisional hernia. In the L-SILTEP approach, to minimize hernia recurrence, clamps are accessed through a lateral incision, enabling better control of the mesh’s lower edge during deflation to prevent curling. Additionally, placing the incision 2 cm above the McBurney or anti-McBurney point effectively avoids the periumbilical or subumbilical scar areas and reduces the risk of incisional infection. Moreover, the lateral abdominal wall muscles remain intact, and the orientation of the three layers of flat muscles differs, lowering the risk of incisional hernia. In our study, no cases of recurrence or incisional hernia were observed. Although this result may be influenced by the study’s small sample size, the abovementioned procedural details likely contributed to this favorable outcome. Literature reports have indicated an increased incidence of incisional hernia following SILS [24, 25], often attributed to the inherent weakness of the umbilical ring in the abdominal wall and improper closure of the umbilical fascia.

The L-SILTEP approach offers numerous benefits. However, it has two limitations. First, it is suitable for unilateral hernia repair but not for bilateral hernias. Second, the skin incision cannot be concealed, making its cosmetic outcome inferior to that of the transumbilical SILS approach [26]. Nevertheless, in this study, 21 patients who underwent open surgery reported that the incision from the SILS approach was significantly more aesthetically pleasing than that from conventional open surgery.

## Conclusion

Preliminary research has shown that the L-SILTEP approach is effective for patients with contraindications to the midline approach and offers some unique advantages. Owing to research limitations such as a small sample size and short follow-up period, further studies are required to validate the safety and efficacy of the L-SILTEP approach.

## Data Availability

Available if requested.
